# Antibacterial Activity of T22, a Specific Peptidic Ligand of the Tumoral Marker CXCR4

**DOI:** 10.3390/pharmaceutics13111922

**Published:** 2021-11-13

**Authors:** Naroa Serna, José Vicente Carratalá, Oscar Conchillo-Solé, Carlos Martínez-Torró, Ugutz Unzueta, Ramón Mangues, Neus Ferrer-Miralles, Xavier Daura, Esther Vázquez, Antonio Villaverde

**Affiliations:** 1 Institut de Biotecnologia i de Biomedicina, Universitat Autònoma de Barcelona, 08193 Cerdanyola del Vallès, Spain; srnaroa@gmail.com (N.S.); josevicente.carratala@uab.cat (J.V.C.); ocs@bioinf.uab.es (O.C.-S.); carlosmartineztorro@gmail.com (C.M.-T.); neus.ferrer@uab.cat (N.F.-M.); Esther.Vazquez@uab.es (E.V.); 2 Departament de Genètica i de Microbiologia, Universitat Autònoma de Barcelona, 08193 Cerdanyola del Vallès, Spain; uunzueta@santpau.cat; 3 CIBER de Bioingeniería, Biomateriales y Nanomedicina (CIBER-BBN), 28029 Madrid, Spain; rmangues@santpau.cat; 4 Biomedical Research Institute Sant Pau (IIB-Sant Pau), Hospital de la Santa Creu i Sant Pau, 08025 Barcelona, Spain; 5 Josep Carreras Research Institute, 08916 Barcelona, Spain; 6 Catalan Institution for Research and Advanced Studies (ICREA), 08010 Barcelona, Spain

**Keywords:** antimicrobial peptides, nanoparticles, fusion proteins, inhibition of biofilm formation, multivalent drugs

## Abstract

CXCR4 is a cytokine receptor used by HIV during cell attachment and infection. Overexpressed in the cancer stem cells of more than 20 human neoplasias, CXCR4 is a convenient antitumoral drug target. T22 is a polyphemusin-derived peptide and an effective CXCR4 ligand. Its highly selective CXCR4 binding can be exploited as an agent for the cell-targeted delivery and internalization of associated antitumor drugs. Sharing chemical and structural traits with antimicrobial peptides (AMPs), the capability of T22 as an antibacterial agent remains unexplored. Here, we have detected T22-associated antimicrobial activity and biofilm formation inhibition over *Escherichia coli*, *Staphylococcus aureus* and *Pseudomonas aeruginosa*, in a spectrum broader than the reference AMP GWH1. In contrast to GWH1, T22 shows neither cytotoxicity over mammalian cells nor hemolytic activity and is active when displayed on protein-only nanoparticles through genetic fusion. Under the pushing need for novel antimicrobial agents, the discovery of T22 as an AMP is particularly appealing, not only as its mere addition to the expanding catalogue of antibacterial drugs. The recognized clinical uses of T22 might allow its combined and multivalent application in complex clinical conditions, such as colorectal cancer, that might benefit from the synchronous destruction of cancer stem cells and local bacterial biofilms.

## 1. Introduction

The peptide T22, also known as [Tyr5,12,Lys7]-polyphemusin II, is a 18-mer derivative of the horseshoe crab cationic antimicrobial peptide (AMP) polyphemusin I, in which three amino acid replacements enable it for a precise binding to the cell surface chemokine receptor CXCR4 [1,2]. Since CXCR4 is an HIV co-receptor [3], T22 was developed as an anti-HIV peptide potentially effective in antiretroviral therapies, blocking the fusion between the viral envelope and the cell membrane and thus preventing viral infection [1,4]. From another point of view and taking advantage of its selective CXCR4 binding, T22 has been largely exploited as a targeting agent, for precision therapies against diverse CXCR4^+^ human cancers. Among others, these include leukemia, lymphoma, head and neck and colorectal cancer [5,6,7,8,9,10,11,12,13,14], in which CXCR4 is overexpressed in metastatic cancer stem cells. In this context, when T22 is engineered as an N-terminal peptide in H6-tagged proteins it promotes, due to its cationic character [15], protein self-assembly into homomeric nanoparticles [16], which include around 10 monomers positioned in a regular, toroidal architecture [17]. The multivalent display of T22 on the particle surface and the nanometric size of these constructs (usually ranging from 12 to 30 nm, depending of the domain composition of the fusion protein) enhances CXCR4 binding and the consequent penetrability into CXCR4^+^ cells, while preventing the renal filtration of chemically coupled drugs [18]. By a combination of all these properties, T22 ensures the architectonic stability of the protein material in the bloodstream and allows a selective intracellular accumulation of T22-empowered protein-only nanoparticles and associated drugs into CXCR4-overexpressing cancer stem cells [17,18,19]. Then, upon the systemic administration of the reporter protein T22-GFP-H6 and derived cytotoxic constructs, a precise in vivo biodistribution is observed, with the destruction of CXCR4-overexpressing cancer tissues and metastatic foci in the absence of side toxicities [18].

Despite polyphemusins generically displaying potent antimicrobial activities [4,20,21,22,23], these functionalities and their interactivity with bacterial cell membranes largely depend on the precise amino acid sequence, which is highly sensitive to even a few amino acid substitutions [24,25]. This is because even point mutations can alter its amphipathicity and hydrophobicity, features that have been postulated to be pivotal in maintaining the right balance between toxicity and antimicrobial activity [26,27,28]. Although several structural variants of polyphemusin peptides have been tested for antimicrobial properties [24,25], T22 has been never explored in this regard. Considering the growing need for new antimicrobial agents and the proved clinical potential of T22 in cell-targeted drug delivery [11,18,29], the detection of any new antimicrobial activities in this peptide would be of broad interest and deserves a thorough investigation. More so, these functionalities might be conserved in T22 peptides displayed on multimeric protein nanoparticles, since antimicrobial activities largely benefit from nanostructured and multivalent presentations [30,31]. Additionally, the combination of anticancer and antimicrobial properties could be of special relevance in many cancers and cancer-linked conditions in which bacteria have a predominant or even triggering role [32,33,34,35,36].

## 2. Material and Methods

### 2.1. Peptides, Proteins and Protein Nanoparticles

T22 (RRWCYRKCYKGYCYRKCRK(5,6-FAM)) was synthetized by Caslo Aps (Caslo Aps Kongens, Lyngby, Denmark) and GWH1 (GYNYAKKLANLAKKFANALWC) by NZYTech (NZYTech, Lisboa, Portugal). An additional C-terminal lysine was included in the synthetic T22 for potential functionalization. On the other hand, production and purification of the fusion proteins T22-GFP-H6 [37], T22-PE24-H6 [38], GWH1-GFP-H6 [39] and GFP-H6 [40] have been precisely described elsewhere. Briefly, these are fusion polypeptides consisting of three functional modules (with the exception of the bimodular construct GFP-H6). In them, the hexahistidine H6 is placed at the carboxy terminus, and the cationic peptides T22 or GWH1 at the amino terminus. The core module is either the enhanced GFP or the de-immunized catalytic domain of *Pseudomonas aeruginosa* exotoxin A (PE24), which displays a potent cytotoxic activity. All proteins were produced by the expression of codon-adapted synthetic genes carried by the expression vector pET22b, in *Escherichia coli* Origami B (BL21, OmpT−, Lon−, TrxB−, Gor−, Novagen, Merck, Darmstadt, Germany), at yields ranging between 3 and 71 mg/L. Proteins were then purified through the H6 domain by Immobilized Metal Affinity Chromatography (IMAC) using a HiTrap Chelating HP 1 mL column (GE Healthcare, Piscataway, NJ, USA), in an AKTA purifier FPLC (GE Healthcare, Piscataway, NJ, USA). The eluted proteins showed a purity level usually over 95%.

### 2.2. Bacterial Growth and Determination of the Minimum Inhibitory Concentration

The effects of the different antimicrobial agents were evaluated against *E. coli* ATCC 25922, *S. aureus* ATCC 29737 and *P. aeruginosa* ATCC-27853. The assay was performed using a broth microdilution method. In 96-well plates, after a two-fold dilution process, each well contained a specific amount of the corresponding peptide, ranging from 2 to 32 µmol/L for GWH1 and 2 to 64 µmol/L for T22 in Mueller Hinton Broth Cation-adjusted medium (MHB-2, Sigma-Aldrich, Saint Louis, MO, USA). Then, 50 µL of MHB-2 containing 10^6^ colony forming units per mL (CFU/mL) was inoculated in each well. After inoculation, the plates were incubated without agitation at 37 °C for 18 h. Bacterial growth was measured by OD_620_. The effect of protein nanoparticles in bacterial growth was analyzed following the same protocol with concentrations ranging from 2 to 16 µmol/L, in *S. aureus* ATCC 29737. Maximal growth was achieved in control wells with no protein and each concentration was evaluated in duplicate. To determine the minimum inhibitory concentration (MIC) of the agents, the lowest concentration showing no bacterial growth, evaluated by visual inspection, was taken. The raw numerical data for all the experimental can be found in the dataset in the Supplementary Materials.

### 2.3. Time-Killing Kinetic Assay

Different concentrations of GWH1 and T22 were distributed in 96-well plates and incubated with a suspension (in Mueller Hinton Broth Cation-adjusted medium, MHB-2, Sigma-Aldrich, Saint Louis, MO, USA) containing 10^6^ CFU/mL of *E. coli* ATCC 25922 or *S. aureus* ATCC 29737. Plates were incubated without agitation at 37 °C. At the indicated times (0, 0.5, 1, 2, 3, 4, 5 and 24 h), an aliquot of 10 μL (out of a total of 200 μL per well) was serially diluted (10-fold) in a different 96-well plate and subsequently seeded in LB plates to evaluate the bacterial viability by CFU counting. Each concentration was evaluated in triplicate and each dilution was seeded in duplicate; therefore, a maximum of six individual counts were used to determine the final CFU for each concentration. A control was included to evaluate bacterial growth in absence of the peptides. 

### 2.4. Evaluation of Biofilm Formation

Biofilms were formed by addition of 106 CFU mL^−1^ of the bacterial suspension (*E. coli* ATCC 25922 or S. aureus ATCC 29737) in sterile, flat-bottomed, 96-well polystyrene micro-well plates (100 μL per well) and incubated in a static condition for 18 h at 37 °C. To determine the antibiofilm activity, different concentrations of the peptides GWH1 and T22 were added to the wells to prevent cell adherence. After incubation, the total biomass of the biofilm was analyzed using the crystal violet (CV) staining method [41]. The contents of the wells were discarded and washed three times with distilled water to remove the planktonic bacteria. Then, biofilms formed by adherent sessile bacteria in the plate wall were fixed by air-drying at 60 °C for 60 min and stained for 15 min with 150 μL of (CV) solution at 0.1%. The stained biofilms were again washed with distilled water and dried for 30 min at 37 °C. Finally, the adhered biofilms were extracted with 200 μL of 30% acetic acid. The biofilm quantification was determined by the photometric measurement of the CV intensity at 550 nm using the multilabel plater Reader VICTOR3 (PerkinElmer, Inc., Waltham, MA, USA). Each concentration was evaluated in duplicate.

### 2.5. Mammalian Cell Viability Assay

The potential cytotoxicity of peptides was tested in murine embryo (NIH3T3 cells) and human lung (MRC-5 cells) fibroblasts and in cervical cell line (HeLa). NIH 3T3 ATCC CRL-1658 cells were maintained in Dulbecco’s Modified Eagle’s Medium (Gibco, Thermo Fisher Scientific, Waltham, MA, USA) and MRC-5 ATCC CCL-171 cells and HeLa cells were maintained in Eagle’s Minimum Essential Medium (Gibco, Thermo Fisher Scientific, Waltham, MA, USA). All cell lines were supplemented with 10% fetal bovine serum (Gibco, Thermo Fisher Scientific, Waltham, MA, USA) and incubated in a humidified atmosphere at 37 °C and 5% of CO_2_. A total of 5000 cells/well for fibroblasts and 3000 cells/well for cervical cells were cultured in opaque-walled 96-well plates for 24 h at 37 °C until reaching 70% confluence and were then exposed to peptides at 8, 16, 32 and 64 μmol/L for 48 h. After incubation, CellTiter-Glo^®^ Luminescent Cell Viability Assay (Promega, Madison, WI, USA) was used to determine the potential peptide cytotoxicity. The luminescent signal, proportional to the amount of ATP present in the sample, was measured in a conventional microplate reader VICTOR3 (PerkinElmer, Inc., Waltham, MA, USA). The cell viability experiments were performed in triplicate.

### 2.6. Hemolysis Assay

Freshly drawn human erythrocytes were harvested by centrifugation for 5 min at 1500 g and washed three times with PBS (137 mM NaCl, 2.7 mM KCI, 10 mM Na_2_HPO_4_, 1.8 mM de KH_2_PO_4_). Subsequently, a work solution was prepared by diluting the washed erythrocytes with PBS (1%, *v*/*v*). In a 96-well conical bottom plate, the 1% (*v*/*v*) erythrocyte suspension was incubated for 1 h at 37 °C with different concentrations (16, 32 and 64 μmol/L of the GWH1 and T22 peptides. After incubation, the plates were centrifuged for 5 min at 1500× *g*, and the supernatant was transferred to a new 96-well plate to measure the absorbance at 405 nm in a multilabel plater Reader VICTOR3 (PerkinElmer, Inc., Waltham, MA, USA). Two controls were included, PBS as a non-hemolysis control and Triton X-100 as a 100% hemolysis control. Experiments were performed in triplicate.

### 2.7. Measurement of the Nanoparticle Size

Size distribution of protein samples was determined by dynamic light scattering (DLS). Average values were obtained after the independent measurement of protein samples in triplicate, at 633 nm, in a Zetasizer Nano ZS (Malvern Instruments Ltd., Malvern, UK). 

### 2.8. Structure-Based Calculations and Molecular Modeling

Polyphemusin-I (PM1; Protein Data Bank –PDB– code: 1RKK) [24], Tachyplesin-1 (TL1; PDB code: 1WO0); http://dx.doi.org/10.2210/pdb1wo0/pdb, accessed on 23 July 2021), Arenicin-3 (AR3; PDB code: 5V0Y) [42]; Protegrin-1 (PG1, PDB code: 1PG1) [43], Gomesin (GM, PDB code: 1KFP) [44], Thanatin (TT, PDB code: 8TFV) [45], PV7 (PM1 synthetic structural variant) [25] and T22 (a polyphemusin II analog) [4] were used as model β-hairpin antimicrobial peptides. For each multimodel NMR structure downloaded from the PDB, the model closest to the average was selected as representative (https://swift.cmbi.umcn.nl/servers/html/bestml.html, accessed on 23 July 2021). Foldx v5 [46] RepairPDB function was applied to all selected structures. PV7 was modeled from PM1 using the BuildModel function from FoldX v5 (Fundació Centre de Regulació Genòmica, Barcelona, Catalonia, Spain), T22 was modeled from PM1 too, PyMOL builder tool (The PyMOL Molecular Graphics System, Version 2.0 Schrödinger, LLC. New York, NY, USA. https://pymol.org, accessed on 4 January 2021) was used to add the extra lysine at the C-terminus and then FoldX v5 RepairPDB and BuildModel functions were applied. The 3D-HM web application (Karlsruhe Institute of Technology, Karlsruhe, Baden-Württemberg, Germany) [47] was used to generate the GWH1 structure as well as to calculate the net charge, average absolute electrostatic potential at the peptide’s surface and Hydrophobic Moment (HM) vector (a representation of the distribution of polar and nonpolar parts in a molecular surface) for all analyzed structures. VMD version 1.9.4a51 [48] and PyMOL version 2.4.2 were used to generate figures with these results. PDB2PQR version 3.1.0 [49] was used to add hydrogen atoms to all structures before submitting them to 3D-HM. PDBparam (https://www.iitm.ac.in/bioinfo/pdbparam/index.html, accessed on 20 July 2021) [50] was used to calculate the surface hydrophobicity and what the authors call hydrophobic free energy (a solvent-accessible-area-based estimate of the non-polar component of the change in solvation free energy upon folding). Superposition of structures for analysis or representation in figures was based on the main chain N, CA, C and O atoms with the McLachlan algorithm [51] as implemented in ProFit v3.3 (http://www.bioinf.org.uk/software/profit/, accessed on 22 March 2021) and using residue equivalences as obtained with jCE, the java implementation of the CE method [52].

## 3. Results

**Figure 1:**
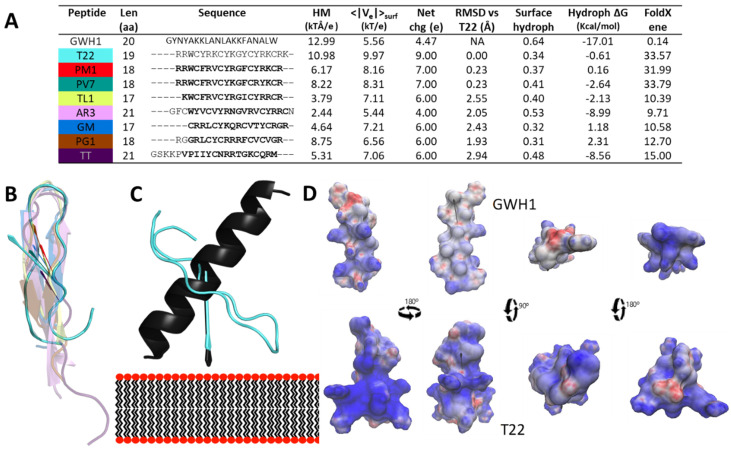
Structure of GWH1, T22 and related AMPs. (**A**) Columns contain the following data: peptide name (β-hairpin-forming peptides with background colored as in (**B**); length in number of amino acids; peptide sequence aligned with T22 (all but GWH1), residues used for superposition in bold; hydrophobic-moment-vector magnitude; average absolute electrostatic potential on the peptide’s surface; net charge; RMSD from T22 superposition; surface hydrophobicity; hydrophobic free energy (see methods for definition); FoldX total energy after repair. (**B**) Superposition of all β-hairpin AMPs used in this study and their calculated HM vectors against model T22 (RMSD and colors for each peptide given in (**A**). (**C**) Superposition of the calculated HM vector for T22 and GHW1, oriented parallel to the membrane normal. (**D**) Representation of the surface electrostatic potential for T22 and GWH1 (scale: top red = −10, top blue = 10).

The generated T22 model reproduces an early NMR structure of T22 [4], which revealed the conservation of the hairpin structure that promotes the interaction with the membrane and antimicrobial activity of PM1 and other AMPs [28]. A closer analysis of the T22 structure (Figure 1) demonstrates a similarity of traits with other AMPs, including length, hydrophobicity, net charge and amphipathicity, as reflected by the Hydrophobic Moment (HM) vector. Although the majority of its properties are naturally closer to those of other β-hairpin-forming AMPs (Figure 1A, all but GWH1), in particular and as expected the close relative PM1 and its derivative PV7 (Figure 1A,B), it stands out that T22 presents an HM magnitude (a quantity that increases with the unbalance of the distribution of polar and nonpolar surface areas in the molecule, i.e., amphipathicity) closest to GWH1. When the corresponding HM vectors are aligned with the membrane normal it is shown that T22 and GWH1 present very similar tilts (Figure 1C, the alignment of the HM vector and the membrane normal provides an indication of the orientation that the peptide may adopt when inserted in the membrane).

**Figure 2:**
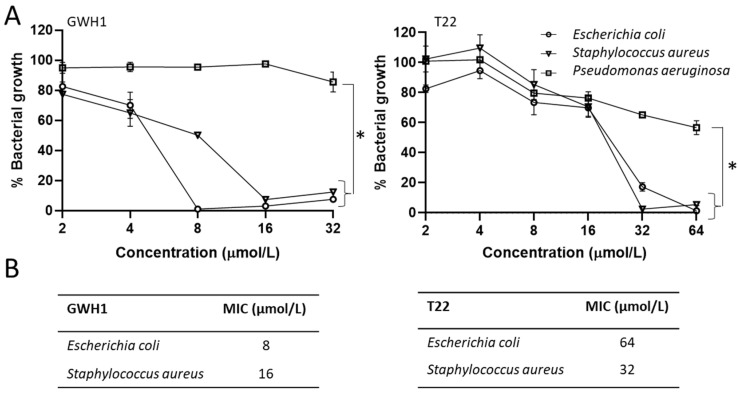
Impact of peptides T22 and GWH1 on bacterial growth in liquid culture. (**A**) Bacterial growth of *E. coli*, S. *aureus* and *P. aeruginosa* cultures, measured by their optical density at 620 nm, treated with T22 and control GWH1 peptides in serial 2-fold dilutions at 37 °C for 18 h. Each point represents an average of at least two different values and error bar indicates standard deviation. (**B**) Minimum inhibitory concentration (MIC) of the peptides for *E. coli* and *S. aureus*. The lowest concentration showing no bacterial growth (evaluated by visual inspection) in the broth microdilution method was taken as the MIC. Significant differences between groups are indicated as * *p* < 0.01, one-way analysis of variance (ANOVA) followed by Tukey’s multiple comparisons test.

**Figure 3:**
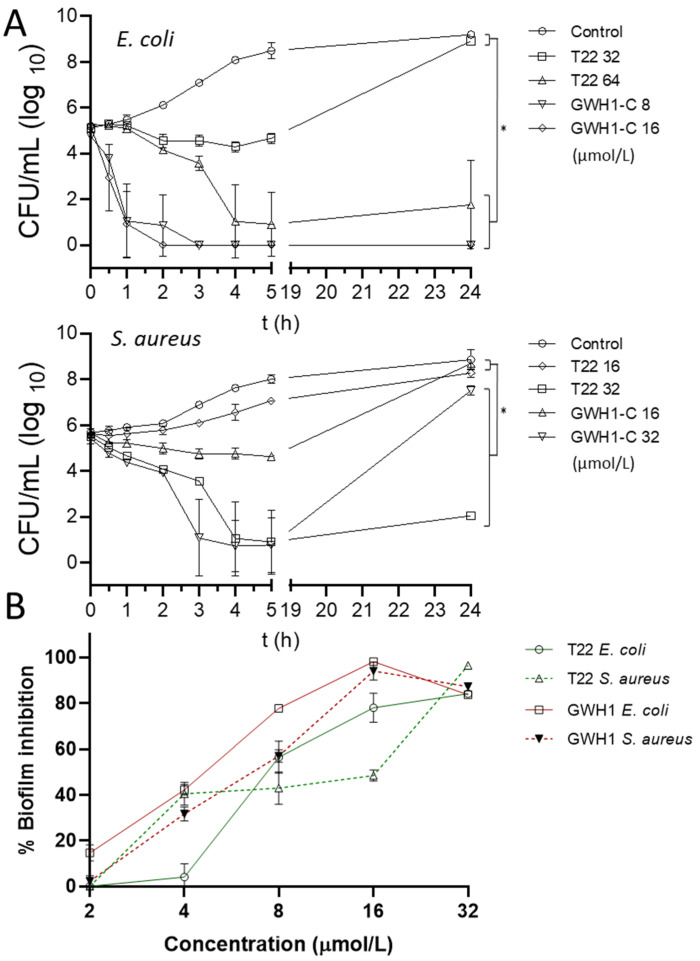
Time- and concentration-dependent impact of the peptides on bacterial growth and biofilm formation, respectively. (**A**) Time-kill kinetics of *E. coli* (up) and *S. aureus* (down) exposed to T22 and GWH1 peptides at different concentrations for 0, 0.5, 1, 2, 3, 4, 5 and 24 h. Each point represents an average of at least two different values and error bars indicate standard deviation. Control represents the bacterial growth without peptide exposure. Figures indicate concentration in µmol/L. (**B**) Effect of peptide concentration on biofilm formation on the surface of the microtiter wells. Significant differences over bacterial control are indicated as * *p* < 0.05, one-way analysis of variance (ANOVA) followed by Tukey’s multiple comparisons test.

In view of these AMP-like physicochemical properties, T22 was tested for its antimicrobial activity over liquid cultures of three bacterial pathogens, namely *Escherichia coli*, *Staphylococcus aureus* and *Pseudomonas aeruginosa*. GWH1, of similar length (Figure 1A), was used as control. GWH1 is a non-natural peptide, developed as AMP [53,54], that adopts an amphipathic helical structure when bound to a membrane and its GFP fusion construct (GWH1-GFP-H6) self-assembles similarly to T22-GFP-H6 [55]. Upon exposure to bacterial cultures, both peptides promoted a clear drop of optical density in a dose-dependent manner (Figure 2A), with *E. coli* and *S. aureus* being the most sensitive species. GWH1 was superior to T22 in terms of its antibacterial activity, with lower MIC values in all cases (Figure 2B). While the biological impact of GWH1 was immediate, T22 required longer times to reach comparable disruptive effects over bacterial cells (Figure 3A,B). In addition, both peptides inhibited biofilms formed by *E. coli* and *S. aureus* (Figure 3B), which we value as a promising feature regarding the potential applicability of T22 as an AMP.

**Figure 4:**
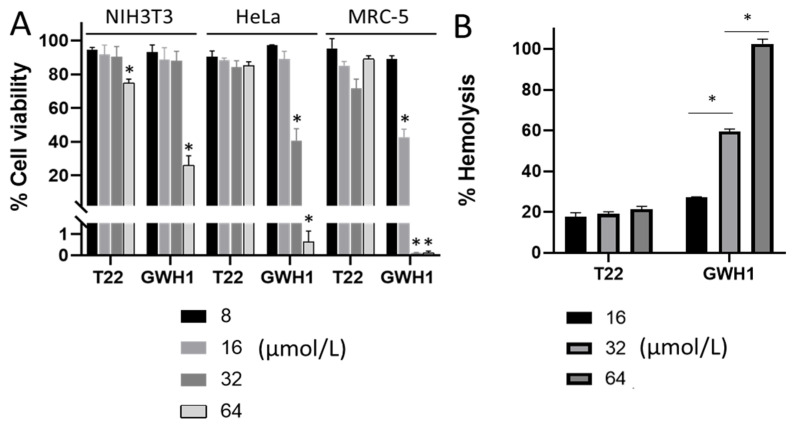
Cytotoxicity of peptides T22 and GWH1 on mammalian cells. (**A**) Cell viability in the presence of peptides over cultured mammalian cells, recorded 48 h after exposure. (**B**) Hemolytic activity associated with peptides over human erythrocytes. Significant differences over cell control are indicated as * *p* < 0.01, one-way analysis of variance (ANOVA) followed by Tukey’s multiple comparisons test. Figures indicate concentration in µmol/L.

Due to their interaction with biological membranes, many AMPs show hemolytic or cytotoxic activities over mammalian cells, bottlenecking a widespread use of these materials as safe drugs [56,57]. In this context and as expected, GWH1 showed a mild cytotoxicity (Figure 4A) and a dose-dependent hemolysis (Figure 4B) that compromise its clinical use. In contrast, T22 shows only a moderate or absent cytotoxicity in several cell lines (Figure 4A) and a complete absence of hemolytic activity up to the very high doses tested here (Figure 4B), which represents a clear competitive advantage over the control peptide GWH1.

If T22 would keep its antimicrobial activities when presented in assembled, protein-only nanoparticles, it would have a potential dual application as a CXCR4-targeting agent and AMP. It is widely recognized that bacterial infections not only represent further complications in solid tumors [58,59,60] but that they also participate in tumor formation or as triggering agents in several human neoplasias [32,33]. Such triggering effects are specially suspected in organs such as colon that are continuously exposed to microbiome components that might largely contribute to, or modulate, the initiation and progression of colorectal cancer [61,62,63,64,65]. Particularly, the formation of *E. coli* biofilms has been recently pointed out as an oncogenic driver in colorectal cancer development [66], and as shown above (Figure 3B), T22 is a good inhibitor of *E. coli* biofilm formation. On the other hand, T22, in the form of fusion proteins assembled as nanoparticles, has proved to be highly effective in the targeted delivery of antitumoral drugs. In this context, the *P. aeruginosa* exotoxin A has been genetically inserted in a T22-based protein construct, thus generating the build-in cytotoxic, CXCR4-targeted nanoparticle T22-PE24-H6. In animal models of metastatic human cancers, T22 confers selectivity for CXCR4-overexpressing cancer stem cells while the bacterial toxin PE24 causes cell death and cancer remission [6,8]. The combination of the toxin and T22 is then a clinically promising concept [18,67]. 

**Figure 5:**
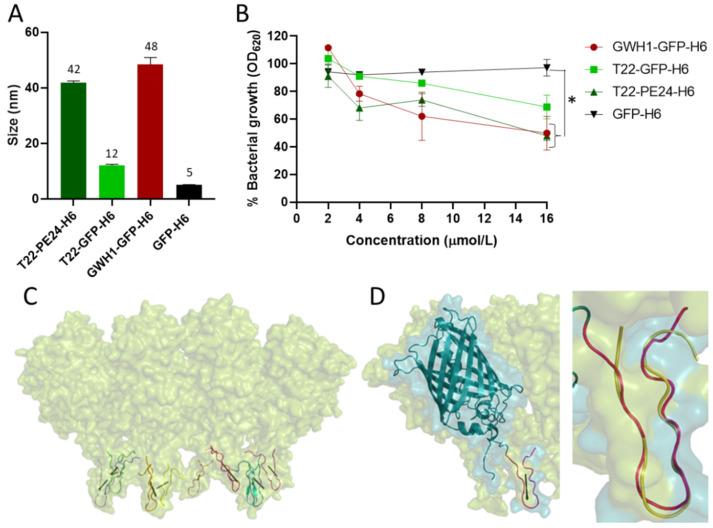
Antibacterial activity and possible structure of a recombinant T22 displayed on protein nanoparticles. (**A**) Volume size distribution of T22-PE24-H6, T22-GFP-H6 and GWH1-GFP-H6 nanoparticles and unassembled GFP-H6, as determined by dynamic light scattering. Data are represented as mean ± standard error on the mean (SEM). (**B**) Bacterial integrity of *S. aureus* measured by the optical density at 620 nm, after incubation of GWH1-GFP-H6, T22-GFP-H6 and T22-PE24-H6 nanoparticles and GFP-H6 in serial 2-fold dilutions at 37 °C for 18 h. Protein concentration at the X axis refers to monomers. Significant differences over GFP control protein are indicated as * *p* < 0.01, one-way analysis of variance (ANOVA) followed by Tukey’s multiple comparisons test. (**C**) A proposed model for the T22-GFP-H6 nanoparticle according to a previous approach [17]. Each T22 peptide has been colored differently and its calculated HM vector has been drawn in black. (**D**) Left: detail of (**C**) for a single nanoparticle monomer. Right: Closeup of the T22 region with PM1-selected model superposed (RMSD 1.34 calculated with PyMOL’s “super” function).

Envisaging a dual role of T22 in protein constructs, the T22-empowered fusions T22-GFP-H6 and T22-PE24-H6, assembled as regular nanoparticles (Figure 5A), were tested for their potential activity as antibacterial agents against *S. aureus* (Figure 5B). Interestingly, the display of T22 in oligomers (Figure 5C) is not only maintained but it also tends to enhance the antibacterial capacity of the free peptide, particularly for T22-PE24-H6 (compare data from Figure 2A and Figure 5B). In the oligomers, 10 copies of the T22 loop are predicted to be exposed to the solvent with their HM pointing in perfect sense and direction to allow interaction with the membrane (Figure 5C). In this orientation, T22 would keep the β-hairpin structure associated with AMP activity (Figure 5D). The observation of AMP activity linked to T22 in the form of multimeric nanoparticles, in which the peptide is genetically fused to the building block, opens interesting routes for the engineering of antimicrobial peptides in more effective formulations easily reachable through simple genetic engineering.

## 4. Discussion

The therapies for solid cancers are based on the resection of the primary tumor and further chemotherapy with cytotoxic, low-molecular weight drugs, administered systemically. The lack of drug targeting is associated with severe side effects [68,69] limiting the usable doses and minimizing the local drug concentration, which usually remains insufficient to prevent recurrence and metastasis [70,71]. Tumor-targeted nanomedicines are pointed out as innovative ways to enhance drug selectivity, increase the local drug concentrations and minimize side effects [72,73,74,75,76,77]. This should result in a higher efficacy at low drug doses, which should concomitantly enhance life quality and survival expectancy. The expression levels of the cytokine receptor CXCR4 are associated with invasiveness and aggressiveness in more than 20 human neoplasias [78,79,80,81,82,83,84,85,86,87,88,89], including colorectal cancer and breast cancer. This makes this cell-surface protein, occurring in metastatic cancer stem cells, a good target for precision therapies [2,10,11,14,84,90,91]. Colorectal cancer is among one of the highly prevalent CXCR4^+^ cancers in men and women, with growing incidence and worldwide spread [92,93,94,95]. Early and advanced lesions in the colon mucosa are endoscopically detected [96,97,98] and resected [97,98,99,100], and the treatment is completed with systemically administered chemotherapy, which exploits the cytotoxic activities of several low-molecular weight drugs such as irinotecan, 5-fluorouracil and capecitabine, among others [101,102,103,104].

T22 assists the protein self-assembly of given constructs in the form of regular protein-only nanoparticles [15,17]. This peptide, as an amino-terminal protein fusion, also targets these constructs to selectively bind and penetrate CXCR4-overexpressing cancer cells [15]. Therefore, we have adapted T22-empowered nanoparticles to deliver Floxuridine [18], Auristatin E [11,29] or several protein toxins [8,13] for the selective destruction of colorectal cancer tissues. In the present study, we have demonstrated that T22 also shows a modest antimicrobial activity (Figure 2 and Figure 3) and a capacity to inhibit biofilm formation broader to that of other conventional AMPs such as GWH1 and over Gram-negative and Gram-positive species. GWH1 and T22 are both amphipathic peptides, that is, with hydrophilic and hydrophobic sides. However, the distribution of charges in their surfaces is completely different, a fact that generates a much clearer difference between sides in GWH1 than in T22 (Figure 1D). The magnitudes of their hydrophobic moment (HM) vectors, a measure of amphipathicity, are in both cases high, which has been correlated with the membrane pore-formation capacity [27], a common feature of AMPs. Amphipathicity, however, is also related to toxicity over mammalian cells [105,106,107]. It has been postulated that a right balance between amphipathicity and hydrophobicity is the key to attaining a high antimicrobial activity and low toxicity [28], although it has also been described that detailed surface electrostatics [108] and the HM angle [47,109] highly influence the outcome. On the other hand, it has been proposed that an HM-vector-magnitude threshold, also modulated by the other factors mentioned, could exist that defines the onset of toxicity [27]. Thus, while T22 and GWH1 share features expected in many AMPs, such as a high HM-vector magnitude and a certain tilt angle relative to the membrane normal, the slightly lower amphipathicity, higher net charge and lower hydrophobicity of T22 may result in the absence of generic toxicity and hemolysis (Figure 4B), while retaining a moderate antibacterial activity (Figure 2) and potent biofilm inhibition capacity (Figure 4). This set of properties would be kept in complex macromolecular structures as long as the peptide remains accessible on the surface (Figure 5).

These results are relevant not only when considering T22 and its fusion construct as an AMP but also the combination of the AMP activity with its functionality as a targeting agent in advanced nanomedicines to treat colorectal cancer. In this type of cancer, chemotherapy upon resection is applied by intravenous infusion. However, a recent study [110] proposes the administration of 5-fluorouracil-loaded nanoparticles against colorectal cancer via intestinal mucosa. The concept of surface chemotherapy of colorectal cancer lesions via gastric administration is supported by independent studies stressing the possibility of mucosal treatment of this type of cancer through various categories of polymeric materials [111,112,113,114,115]. In contrast to systemic administration, such an approach would allow, using appropriate agents, a combined treatment to kill cancer cells and concomitantly inhibit participating bacterial biofilms at the local level. If tumor cell-targeted, such a dual treatment would have localized effects on damaged mucosal areas with enhanced precision and effectiveness. Since attaching small chemical drugs to T22-empowered protein-only nanoparticles does not interfere with the targeting abilities of this peptide [29,55], the set of findings presented here opens a new line of exploratory research addressed to combine targeted drug delivery and overlapping biofilm inhibition at the local level through the mucosal delivery of nanoparticles [110]. This possibility is exemplified here by the pleiotropic character of T22 as a structural agent for nanoparticle formation, as a targeting agent and as an AMP.

These notions are relevant in the context of the expanding recognized roles of bacterial biofilms in colorectal cancer development and progression [66,116,117], and regarding the progressive identification of involved bacterial species and consortia [118], which highlights *E. coli* as one among the relevant contributor species through several virulence factors [66,119,120]. Importantly, and due to a distinctive microbiota composition [121,122,123], biofilm formation appears to be specifically relevant in the right-sided colorectal cancer [121], for which antibiofilm drugs could be specially effective. As the microbiota components involved cancer development and progression are more precisely identified, highly focused studies about the potency and real efficacy in vivo of dual-acting protein agents such as T22 should be conducted. 

## 5. Conclusions

A biologically significant antimicrobial activity with associated biofilm destruction has been found, for the first time, associated with T22, a short peptide used to selectively target nanomedicines for CXCR4^+^ human cancers. Such activity is maintained in the nanostructured forms of the peptide, ideal for drug delivery, in the absence of toxicity over human cells. Even being moderate, the antibacterial capacity of T22 is superior to that shown by other reference antimicrobial peptides. This discovery and the supporting general concepts open the possibility to design nanomedicines for human neoplasias, such as colorectal cancer, that show an important bacterial component. In this context, a local dual performance combining the selective (or even broad) antimicrobial impact and highly selective antitumoral activity could be extremely interesting as a new way to develop more effective, multifunctional anticancer therapies or preventive approaches from the mucosal side of the tumor that might complement the currently applied systemic therapies.

## Data Availability

Data is available at Table S1.

## Supplementary Materials

The following are available online at https://www.mdpi.com/article/10.3390/pharmaceutics13111922/s1, Table S1: Raw Data.
